# Isolation and cloning of the endoglucanase gene from *Bacillus pumilus* and its expression in *Deinococcus radiodurans*

**DOI:** 10.1007/s13205-013-0127-3

**Published:** 2013-03-21

**Authors:** Sachin Telang, Poonam Patel, Vishwas Sarangdhar, Sheela Donde

**Affiliations:** 1 Department of Life Science and Biochemistry, Caius Research Laboratory, St. Xavier’s College, Mumbai, 400001 India; 2 Caius Research Laboratory, St. Xavier’s College, Mumbai, 400001 India; 3 Present Address: Indian Institute of Science Education and Research (IISER), Sai Trinity Building, Sutarwadi Road, Pashan, Pune, 411021 India

**Keywords:** *Bacillus pumilus*, Endoglucanase, groESL, pRAD1

## Abstract

With the aim of engineering a strain of bacteria that could be used for bioremediation of cellulosic waste in radioactive environments, the gene for the secreted endoglucanase enzyme of *Bacillus pumilis* was decided to be cloned into the radiotolerant bacterium, *Deinococcus radiodurans*. The endoglucanase gene from *B. pumilus* was PCR amplified and cloned into *Escherichiacoli* DH5α using a pDrive vector. It was subsequently sub-cloned into *E.coli*–*Deinococcus* shuttle vector pRAD1 downstream of the *Deinococcus* heat-shock promoter, groESL, and the construct was inserted into *D. radiodurans*. Functional endoglucanase enzyme was expressed in both *E.coli* and *D.radiodurans*.

**Electronic supplementary material:**

The online version of this article (doi:10.1007/s13205-013-0127-3) contains supplementary material, which is available to authorized users.

## Introduction

In natural environments, a large proportion of organic waste is found in the form of cellulosic plant material which accounts for more than half of the total organic carbon, with an annual net yield estimated at 10^11^ tons (Sukumaran et al. 2005; Lynd et al. 2002). Micro-organisms in the soil degrade this cellulose by producing cellulose digesting enzymes like lignocellulases, cellulases, etc. However, laboratories, hospitals and industries which use radioactivity for research, health care or commercial purposes, produce several thousand tons annually of radioactively contaminated solid cellulosic waste, in the form of filter paper, cloth, etc. (Rao 2001). For biodegradation of such cellulosic waste in radioactive environments it is necessary to use radio-tolerant micro-organisms that can produce cellulases.

Bacteria of the genus *Deinococcus* exhibit an extraordinary ability to tolerate and survive in tremendously high levels of radioactivity. This bacterium can not only survive acute exposures to gamma radiation that exceeds 1,500 krad without lethality or induced mutation, but also grow continuously in the presence of chronic radiation without any effect on its growth rate or ability to express cloned genes (Battista 1997). *Deinococcus radiodurans* has been genetically engineered and used for bioremediation of organic pollutants and metals in mixed radioactive environments (Appukuttan et al. 2006; Brim et al. 2003; Daly 2000). Shuttle vectors have also been developed for *D.radiodurans* (Meima et al. 2001). However, *D.radiodurans* has not been exploited to its full potential. This can be attributed to its fastidious growth requirements (Meima et al. 2001).

Our aim was to genetically engineer *D.radiodurans* so that it could be used for bioremediation of cellulosic waste in radioactive environments. We isolated a *Bacillus pumilus* strain from rumen fluid, which produced extracellular endoglucanase enzyme with a broad range of thermal and pH tolerance. We report here the cloning of the endoglucanase gene from this strain of *B. pumilus* into *Escherichia coli,* and its subsequent cloning and expression in *D. radiodurans*.

## Materials and methods

### Bacterial strains and growth conditions

*Bacillus pumilus*, producing large amounts of extracellular cellulases, was isolated in our laboratory from rumen fluid, and identified biochemically at Institute of Microbial Technology (IMTECH, India). *D. radiodurans* R1 was a kind gift from Dr. Daly (2000). *E. coli* DH5α (kind gift from Dr. Shobhona Sharma, TIFR) was grown in Luria–Bertani (LB) liquid medium at 37 ± 1 °C under agitation (180 ± 5 rpm), and growth was assessed by measuring OD_600_. Plasmid DNA isolation from *E. coli* cells and transformation of *E. coli* were carried out as described in Sambrook and David (2001).

*Deinococcus radiodurans* was grown in TGY (1 % Bactotryptone, 0.1 % glucose, and 0.5 % yeast extract) liquid medium at 32 ± 1 °C under agitation (180 ± 5 rpm). Transformation of *D. radiodurans* was carried out as described in Meima et al. (2001) and the transformants were selected on media containing 3 μg/ml of chloramphenicol. Growth and gene expression characteristics of the engineered *Deinococcus* were studied in TGY and rich defined medium (RDM) (Holland et al. 2006) containing either no carbon source (NC) as control or glucose (G) or carboxymethyl cellulose (CMC) as carbon source or a mixture of glucose and CMC (GC).

### Plasmids and primers

Plasmid pDrive was purchased from Qiagen. Plasmid pRAD1 was a kind gift from Dr. Lidstrom (2000). The primers used for cloning and DNA sequencing were obtained from Sigma-Aldrich Chemicals. PCR amplification was carried out using*Taq* polymerase (Fermentas) or Phusion flash™ polymerase (Finnzymes). PCR products were purified using QIAquick PCR purification kit (QIAGEN). All restriction enzymes and ligases were obtained from Fermentas. DNA sequencing was carried out by Enzene Biosciences Pvt. Ltd., Bangalore, India. The various plasmids and primers used in this study are listed in Table 1.

**Table 1:** Strains, plasmids and primers

Strains	Genotype	Source or reference
*E. coli*DH5α	F^−^*recA41 endA1 gyrA96 thi*-*1 hsdR17* (rk^−^mk^+^) *supE44 relAlacU169*	Dr. Shobhona Sharma, TIFR
*B. pumilus*	Wild type	Caius Research Lab.
*Deinococcus radiodurans R1*	Wild type	Daly (2000)
Plasmids	Description	
pDrive	TA-cloning vector	Qiagen
pRAD1	*E. coli*–*D. radiodurans* shuttle vector	Meima and Lidstrom (2000)
pCRLPD	pDrive carrying *B. pumilus* endoglucanase gene	This study
pCRLGPD	pDrive carrying groESL promoter upstream of *B.* *pumilus* endoglucanase gene	This study
pCRLG1D	pDrive carrying groESL promoter	This study
pCRLGPR	pRAD1 carrying groESL promoter upstream of *B.* *pumilus* endoglucanase gene	This study
pCRLGSPR	pRAD1 carrying groESL promoter followed by the *Deinococcus* signal sequences upstream of *B.* *pumilus* endoglucanase gene	This study
Primers	Sequence 5′ to 3′	
Dag-XF	GCCTCTAGACATGTTCAG	This study
Dag-NR	GGTTTCAGCATATGGGGT	This study
DagSF	CAGTGACCTGCAGGCATGTTCAG	This study
DagBNR	GCCTAAAGGTGGATCCATATGGGGT	This Study
Endo-NF	GCGACATATGCACATTTTTGAAACACGC	This study
Endo-ER	ACATCCGAATTCTTATTTATTCGGAAGC	This study
GS1	AGAGAGAACAGCAAGAGAAATCTTTTTCATCGGCAGTGCTCCTGGCATCTGGGGTCCTCCTGTGAG	This study
SP2	CTTGCTGTTCTCTCTCTGACCACGCTGCTCGCGGCCATAGATCTCCATATGCACATTTTTGAAACACG	This study
M13/pUC sequencing primer	CGCCAGGGTTTTCCCAGTCACGAC	NEB
M13/pUC reverse sequencing primer	AGCGGATAACAATTTCACACAGGA	NEB
P5	GGAGCGGATAACAATTTCACACA	Appukuttan et al. (2006)
P6	AACGCGGCTGCAAGAATGGTA	

### Isolation and characterization of a cellulose digester

Rumen microorganisms showing efficient cellulose digestion were selected and purified by repetitive and successive streaking on cellulose agar plates/CMC agar plates and nutrient agar plates. Twelve cellulose digesters from rumen fluid, capable of growing on both Whatman filter paper and CMC, under aerobic condition at 37 °C at pH 6.0 were isolated. One of these isolates showing maximum endoglucanase activity was identified as *B.pumilus* at IMTECH, India.

Characterization of endoglucanase produced by *B.pumilus* was carried out using supernatant of culture grown at 37 °C in CMC broth. Supernatant was collected by centrifuging the broth at 8,000 rpm for 10 min. The activity of endoglucanase on CMC was estimated, by measuring the amount of reducing sugar produced at the end of the reaction, using the DNS method (Miller 1959). The optimum temperature and pH for this enzyme were also determined.

### Cloning of *B. pumilus* endoglucanase gene and *D. radiodurans* groESL promoter in pRAD1

**Fig. 1:**
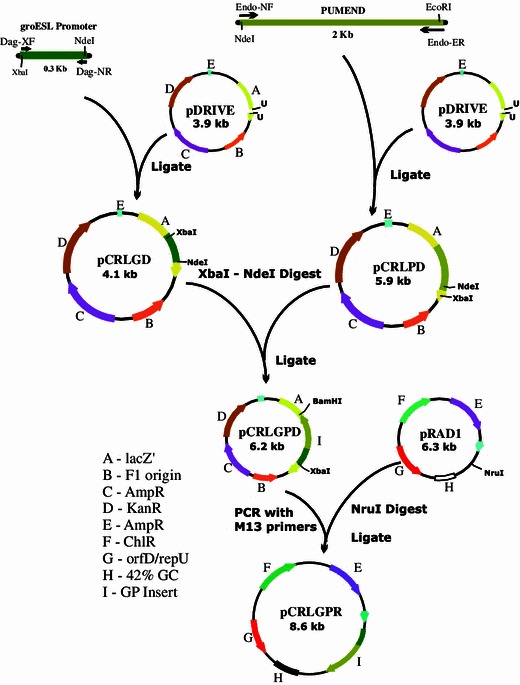
Construction of pCRLGPR

*Bacillus pumilus* endoglucanase gene was cloned into pRAD1 as per the schematics given in Fig. 1. Briefly, the *B. pumilus* endoglucanase gene (PUMEND) (GenBank accession #AF206716.1.) was amplified from the genomic DNA of *B. pumilus* using primers EndoNF and Endo-ER and was cloned into the pDrive TA-cloning vector to give pCRLPD (Caius Research Lab PUMEND pDRIVE). Similarly, groESL promoter (G) was amplified from the genomic DNA of *D. radiodurans* using primers Dag-XF and Dag-NR and cloned into the pDrive vector to give pCRLGD. The groESL promoter from pCRLGD was amplified using M13 primers, digested with XbaI-NdeI and ligated to XbaI-NdeI-cut pCRLPD. Screening for the groESL promoter in-frame with endoglucanase gene (GP cassette) was done by colony PCR using cross primers i.e., Dag-XF and Endo-ER. The GP cassette was amplified from the plasmid construct pCRLGPD by Phusion flash™ using Dag-XF and Endo-ER primers and subcloned into NruI-cut pRAD1, and the resultant plasmid was called pCRLGPR (Caius Research Lab gro PUMEND pRAD1). Presence of GP cassette in pRAD1 was detected by colony PCR with primers P5 and P6.

### Cloning of the *D. radiodurans* signal sequence to give pCRLGSPR

A search was carried out for *D. radiodurans* secreted proteins in the UniProt proteome Knowledgebase to identify a signal peptide specific for *Deinococcus*. A *B.* *subtilase*-type serine protease, DR_A0283, accession number Q9RYM8, was identified to be a potential secreted protease with a 22-amino acid signal sequence (Joshi et al. 2004). This signal sequence was analyzed in silico by the SignalP program (Bendtsen et al. 2004) for prediction of its suitability as a signal peptide, and was found to be a suitable candidate for *Deinococcus*.

The signal peptide used and its corresponding DNA sequence are given below.

**Fig. 2:**
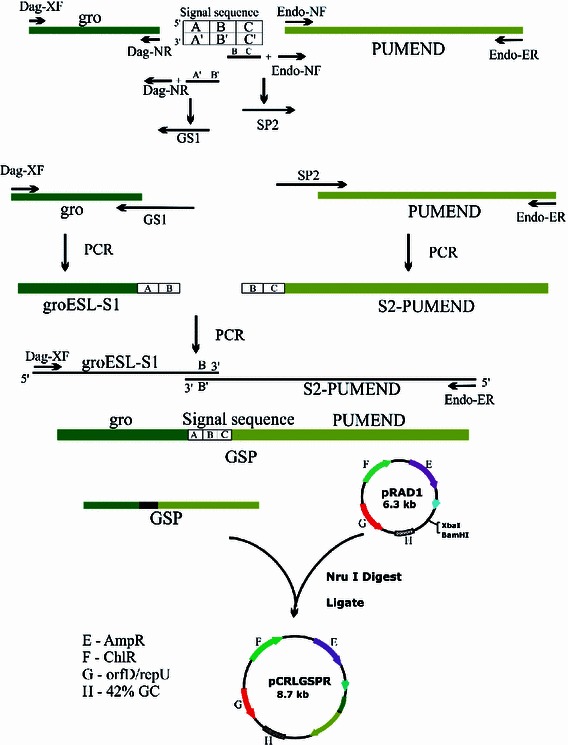
Cloning of the signal sequence to give pCRLGSPR

The steps for inserting the signal sequence between the groESL promoter and PUMEND gene in pRAD1 are shown in Fig. 2. Fusion of groESLS1 and S2-PUMEND was carried out by overlap extension, using a mixture of groESL-S1 and S2-PUMEND as the template DNA. Amplification was carried out using Phusion flash™. The 2.3 kb PCR product, called GSP, contained the signal sequence in-frame with the PUMEND sequence (as shown by sequencing data) and downstream of groESL promoter. This PCR amplicon was ligated to NruI-cut pRAD1 vector to obtain pCRLGSPR. The PUMEND gene sequence from this construct has been submitted to GenBank (Accession # JN681277).

### Expression of endoglucanase gene in *D. radiodurans*

*Deinococcus radiodurans* bearing pCRLGPR was grown at 32 ± 1 °C under agitation, on rich defined medium (RDM) (Holland et al. 2006) containing either glucose (G), CMC (CMC), glucose plus CMC (GC) and no carbon source (NC). *D. radiodurans* bearing pRAD1 was used as a control. Aliquots were collected at 0, 24, 72 and 240 h of growth. After centrifugation of each aliquot, the resulting supernatant and pellet were separately analyzed for endoglucanase activity by the Congo red plate assay (Teather and Wood 1982). Butanol treatment was used to release intracellular enzymes from the cell pellets. Cells were harvested by centrifuging 1 ml culture samples at 16,000×*g* for 2 min. The pellets were resuspended in 250 μl of 0.2 M acetate buffer pH 5.6 supplemented with 50 μl of *n*-butanol. The suspensions were incubated for 5 min at room temperature, centrifuged at 16,000×*g* for 5 min and 10 μl aliquots of the aqueous supernatants were spotted on CMC agar plates for carrying out the Congo red plate assay.

## Results

### Isolation of *B. pumilus* and characterization of its endoglucanase enzyme

**Fig. 3:**
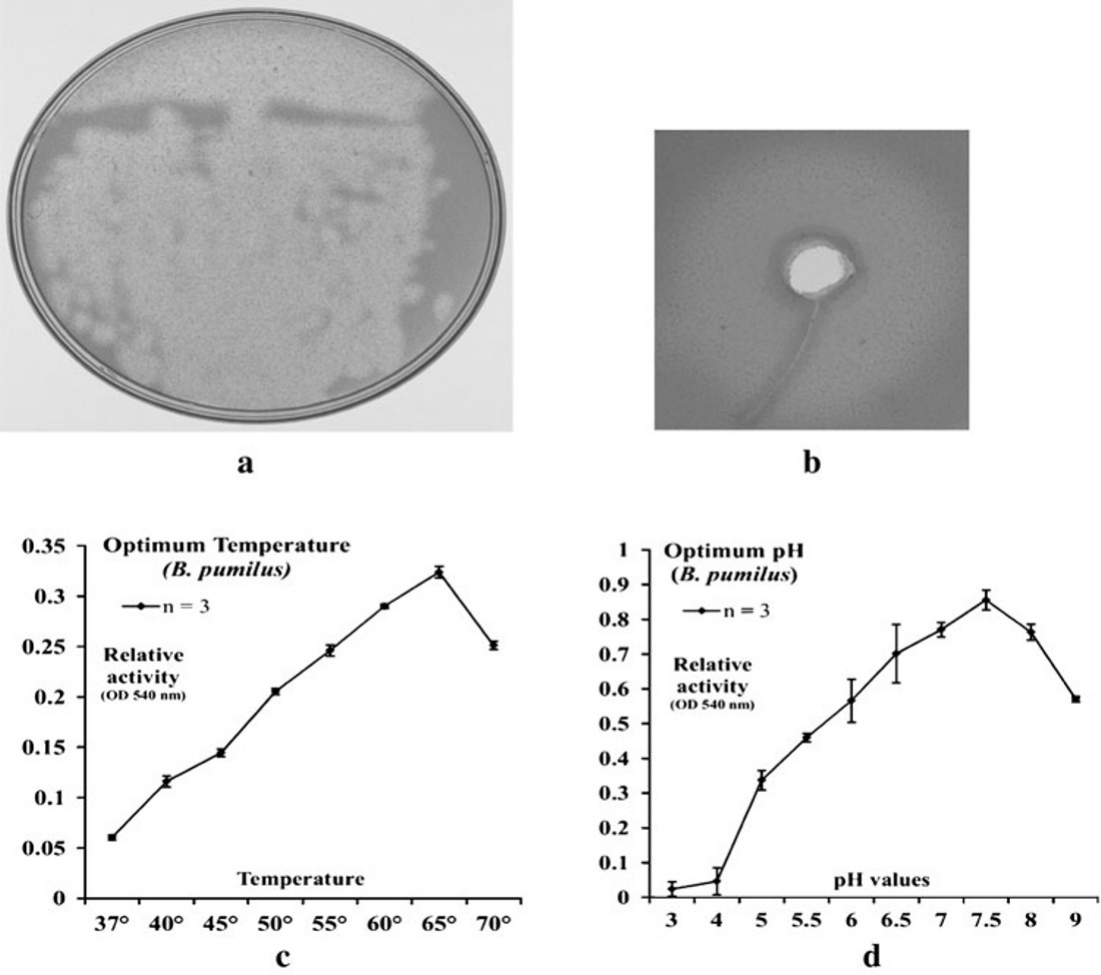
**a** Congo red plate assay showing zones of digestion of CMC around colonies of *B.pumilus* after 24 h growth. **b** Congo red assay of supernates of 24 h old *B. pumilus* culture on CMC plate (using agar well diffusion method). **c** Optimum pH of endoglucanase. **d** Optimum temperature of endoglucanase

Endoglucanase activity of the isolate was confirmed by Congo red plate assay as seen in Fig. 3a, b. The clearance zone on the CMC plate indicates degradation of CMC. The optimum temperature for the secreted endoglucanase of *B. pumilus* was found to be 65 °C and optimum pH was 7.5 as shown in Fig. 3c, d.

The strain *B. pumilus,* which we isolated in our laboratory from rumen fluid, showed CMC digesting ability as seen from the zones of clearing around the colonies and around the culture supernatant. The secreted enzyme also showed activity over a broad range of pH (5–9) and temperature (45–70 °C).

### Restriction digestion of constructs and expression of cloned endoglucanase gene in *E.* *coli*

**Fig. 4:**
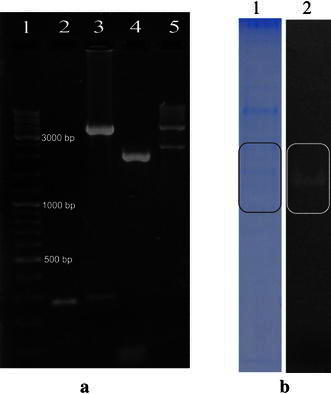
**a** Electrophoretic analysis of plasmid constructs. The amplicons and restriction enzyme-digests of plasmid constructs were separated on a 0.8 % agarose gel in 0.5× TBE. *Lane 1* 100 bp-DNA ladder, *lane 2* groESL promoter amplicon, *lane 3* Xba-*Bam*HI Digest of pCRLGD, *lane 4* Pumend amplicon, *lane 5* Xba-*Bam*HI digest of pCRLPD. **b** Coomassie Blue-stained cell lysate of *E. coli* clone bearing pCRLGPD and its zymogram showing digestion of CMC

We placed this PUMEND sequence downstream of *D. radiodurans* promoter groESL in pDrive vector (pCRLGPD) and confirmed the synthesis of functional endoglucanase enzyme in *E. coli* (as shown in Fig. 4b).

### Expression of cloned endoglucanase gene in *D. radiodurans*

**Fig. 5:**
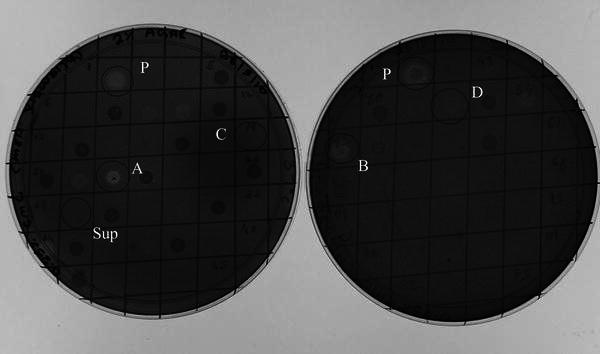
Congo red plate assay of supernatants and pellet extracts of cultures at different time points during growth. ‘*P*’ *B. pumilus* culture supernatant (positive control), ‘*A*’ 24 h butanol-treated GPR pellet, ‘*B*’ 240 h butanol-treated GPR pellet, ‘*C*’ 24 h butanol-treated pRAD1 pellet, ‘*D*’ 240 h butanol-treated pRAD1 pellet, ‘*Sup*’ 24 h GPR supernatant

*Deinococcus radiodurans* bearing pCRLGPR expressed the endoglucanase gene and a functional enzyme was synthesized within 24 h as seen by the clearance zone (Fig. 5 ‘A’), whereas *D.* *radiodurans* bearing pRAD1 (negative control) did not show any enzyme activity even up to 240 h (Fig. 5 ‘C’). However, Endoglucanase enzyme was not detected in the culture supernatant of pCRLGPR (Fig. 5 ‘Sup’). This indicated that the cloned endoglucanase gene was expressed by the engineered *Deinococcus,* but the enzyme was not secreted into the medium.

### Comparison of growth curves of *D. radiodurans* clones

*Deinococcus radiodurans* bearing pRAD1, pCRLGPR and pCRLGSPR were separately inoculated into the following media: (A) TGY (TGY), (B) RDM with glucose (G), (C) RDM with glucose and CMC (GC), (D) RDM with CMC (CMC) and (E) RDM with no additional carbon source (NC), and growth was measured at 600 nm.

**Fig. 6:**
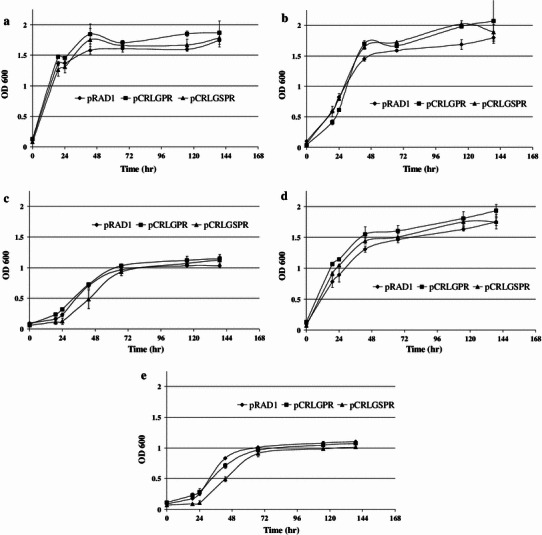
Growth of *D. radiodurans* with constructs grown in the following media: **a** TGY, **b** RDM + glc (G), **c** RDM + CMC (C), **d** RDM + glc + CMC (GC), **e** RDM (NC) (standard error bars are shown *n* = 3)

The growth curves in Fig. 6 demonstrate that *D. radiodurans* with pRAD1, pCRLGPR and pCRLGSPR grew to similar extent in RDM. In RDM containing glucose as the additional carbon source, all three cultures showed higher final OD_600_, and in RDM containing CMC, all three cultures grew to the same extent as in RDM alone.

## Discussion

The endoglucanase gene of *B. pumilus* isolate was PCR-amplified using consensus primer sequences found in closely related bacteria, the resultant amplicon, PUMEND, did not show the presence of a signal sequence. When the sequence was analyzed by the SecretomeP program (Bendtsen et al. 2005), it was predicted that the encoded endoglucanase enzyme could be secreted through the non-classical pathway by *B. pumilus*.

We cloned the GP cassette into pRAD1 vector of *D.* *radiodurans* (pCRLGPR), and showed that this construct could produce functional endoglucanase enzyme in *D. radiodurans* (as shown in Fig. 5), but the enzyme remained intracellular. To make *D.radiodurans* secrete the enzyme; we decided to clone a signal peptide native to *D.radiodurans* upstream of the endoglucanase gene. UniProt Knowledgebase search for *D.radiodurans* secretory proteins revealed a serine protease as one of the ‘putative’ secreted proteins. We introduced the putative signal peptide sequence of this protease of *D.radiodurans* at the N-terminus of the *B. pumilus* endoglucanase gene sequence (PUMEND) and downstream of the groESL promoter, to get pCRLGSPR, and introduced this construct into *D. radiodurans*. Sequencing data confirmed the integrity of the signal sequence in-frame with the downstream cloned endoglucanase gene.

All *D. radiodurans* clones, bearing pRAD1, pCRLGPR or pCRLGSPR achieved a similar growth rate, and a higher final OD_600_ in Rich Defined Medium (RDM) containing glucose (Fig. 6b, d) as compared to growth in RDM (Fig. 6e) indicating lack of secretion of the expressed endoglucanse enzyme. The lack of secretion could be due to the incompatibility between the secretory systems of the two organisms, *Bacillus* and *Deinococcus,* or differences in the complexities of their cell walls (Karrenberg 1985; Bohnsack and Schleiff 2010).

We have successfully cloned the *B. pumilus* endoglucanase gene into *D. radiodurans* and showed expression of functional endoglucanase enzyme in *Deinococcus*, thus achieving the first step towards developing an engineered organism capable of bioremediation of cellulosic waste in radioactive environments. We suggest that a better understanding of the protein secretory mechanism(s) in *D.radiodurans* would be necessary to achieve secretion of the endoglucanase enzyme by this organism.

## Electronic supplementary material

Below is the link to the electronic supplementary material.

Supplementary material 1 (DOCX 15 kb)

Supplementary material 2 (PDF 79 kb)
