# Supplementary material for: Isolation and cloning of the endoglucanase gene from Bacillus pumilus and its expression in Deinococcus radiodurans
Source: 3 Biotech. 2013 Mar 21;4(1):57–65. doi: 10.1007/s13205-013-0127-3 (PMC3909571; doi:10.1007/s13205-013-0127-3)
Supplement: Supplementary file 1 — Supplementary material 1 (DOCX 15 kb) [file 13205_2013_127_MOESM1_ESM.docx]

Supplementary Data

*Bacillus pumilus* endoglucanase gene sequence (PUMEND) (GenBank accession # JN681277)

**1 atgcacattt ttgaaacacg cttgattctt ttcaacactg tacaaaacac gaacggaata**

**61 acaaggaggt ggtcatttct tgttcaatgt ttcacgttta agaaaaaaga aggggtgaga**

**121 agtaggtaca tgtcagatta caactatgta gaggtgcttc aaaaatccat tttgttttac**

**181 gaagcccagc gttccggaaa gcttcctgaa agcaatcgtc ttaactggcg aggggattct**

**241 ggactagagg atgggaaaga tgttggtcat gatttaacag gaggctggta cgatgctggt**

**301 gatcatgtga agttcggact tccgatggct tactcggcag ccgtgcttgc atggacagtc**

**361 tatgagtacc gagaagctta cgaagaggca gagctgcttg atgatatgtt agatcaaatc**

**421 aagtgggcaa ccgattattt tttgaaagcc catacaggtc caaatgaatt ttgggcacaa**

**481 gtaggcgatg ggaacgcgga tcatggctgg tggggtccag cagaagtgat gccgatgaac**

**541 cggccggcat ttaaaattga tggacattgt ccaggaacag aagtagccgc ccaaaccaca**

**601 gccgctttag cagcaggttc aattattttt aaagaaactg atgcgcctta tgcagcaaag**

**661 cttctcaccc atgcaaaaca gctttatgca tttgctgacc aatatcgcgg tgagtataca**

**721 gattgtgtca ccaatgcgca gccattttat aactcttgga gtggctatat tgatgagctc**

**781 atttggggtg gaatatggct ctacttggcg acaaatgacc agacctattt aaacaaagca**

**841 ctaaaagcag tagaagaatg gccgaaggat tgggattata cgtttacgat gtcgtgggac**

**901 aatacctttt ttgcttcgca aattttactt gcgaggatca caaaggagaa aaggtttatc**

**961 gaatcgacag agcgtaacct cgattactgg tcgacgggtt ttgttcaaaa tggaaaagta**

**1021 gaaagaatca cttatacgcc tggcggacta gcgtggttag atcaatgggg gtcacttcgt**

**1081 tatacagcaa atgctgcatt tttagcgttt gtgtacgcag attgggtctc tgatcaagaa**

**1141 aaaaagaatc gataccaaac gtttgcgatc aggcaaacac actatatgtt aggggataat**

**1201 ccgcaaaata gaagctatgt cgttgggttt ggcaaaaatc cgccgatgca tccacaccat**

**1261 cgaactgcac atggctcatg gtctaatcag ctgacaactc cttcttctca tcggcacacg**

**1321 ctttatggag cgcttgttgg ggggcctaat gcgcaggatc agtataccga tgacatctct**

**1381 gactatgcat caaacgaggt agcaacagac tataatgccg cctttactgg aaatgtagcc**

**1441 aaaatggtgc agttgtttgg tcaggggcag tcaaagctgc cgaattttcc gcctaaagaa**

**1501 aaagtggaag atgagttttt tgtagaggca gctgtcatga gtaacgatac aacatctact**

**1561 cagatcaaag ccattctata caatcgatca ggctggccgg caagaagtag tcaatcactt**

**1621 tcttttagat attatgtcaa tctaagtgag atatttgcga agggattcac tgataaagat**

**1681 attcaagtga cagctgttta caatgaaggc gcttccttaa ctccgttaac ggtgtatgac**

**1741 gcatcatccc atatctattt tacagaaatc gattttactg gcgtagctat ttttccagga**

**1801 ggcgaatcgc ttcataagaa ggaaatacag ttccggttat ctgcgccaaa tggtgcgaat**

**1861 atatgggatg cctcaaatga ttattccttt caaggattaa catccaatat gcagaaaaca**

**1921 gcgagaattc ctgtttttga tcaaggtatt ttagcatttg gtacgcttcc gaataaataa**
