# Supplementary material for: Isolation and cloning of the endoglucanase gene from Bacillus pumilus and its expression in Deinococcus radiodurans
Source: 3 Biotech. 2013 Mar 21;4(1):57–65. doi: 10.1007/s13205-013-0127-3 (PMC3909571; doi:10.1007/s13205-013-0127-3)
Supplement: Supplementary file 2 — Supplementary material 2 (PDF 79 kb) [file 13205_2013_127_MOESM2_ESM.pdf]

CLUSTAL 2.1 MULTIPLE SEQUENCE ALIGNMENT

File: M:/Dropbox/Paper/Re paper 3rd/3 Biotech/revisedmanuscript/Resubmission/sequence alignment data Date: Wed Feb 13 23:01:35 2013


```
            ***********************************************************.******************************************************************************************
JN681277    ATGCACATTTTTGAAACACGCTTGATTCTTTTCAACACTGTACAAAACACGAACGGAATAACAAGGAGGTGGTCATTTCTTGTTCAATGTTTCACGTTTAAGAAAAAAGAAGGGGTGAGAAGTAGGTACATGTCAGATTACAACTATGTA   150
AF206716.1  ATGCACATTTTTGAAACACGCTTGATTCTTTTCAACACTGTACAAAACACGAACGGAATGACAAGGAGGTGGTCATTTCTTGTTCAATGTTTCACGTTTAAGAAAAAAGAAGGGGTGAGAAGTAGGTACATGTCAGATTACAACTATGTA   150
            1.......10........20........30........40........50........60........70........80........90.......100.......110.......120.......130.......140.......150

            ***********************************************************************************************************************************.******************
JN681277    GAGGTGCTTCAAAAATCCATTTTGTTTTACGAAGCCCAGCGTTCCGGAAAGCTTCCTGAAAGCAATCGTCTTAACTGGCGAGGGGATTCTGGACTAGAGGATGGGAAAGATGTTGGTCATGATTTAACAGGAGGCTGGTACGATGCTGGT   300
AF206716.1  GAGGTGCTTCAAAAATCCATTTTGTTTTACGAAGCCCAGCGTTCCGGAAAGCTTCCTGAAAGCAATCGTCTTAACTGGCGAGGGGATTCTGGACTAGAGGATGGGAAAGATGTTGGTCATGATTTAACAGGCGGCTGGTACGATGCTGGT   300
            .......160.......170.......180.......190.......200.......210.......220.......230.......240.......250.......260.......270.......280.......290.......300

            ***************************** ************************************************************************************************************************
JN681277    GATCATGTGAAGTTCGGACTTCCGATGGCTTACTCGGCAGCCGTGCTTGCATGGACAGTCTATGAGTACCGAGAAGCTTACGAAGAGGCAGAGCTGCTTGATGATATGTTAGATCAAATCAAGTGGGCAACCGATTATTTTTGAAAGCC   450
AF206716.1  GATCATGTGAAGTTCGGACTTCCGATGGCCTACTCGGCAGCCGTGCTTGCATGGACAGTCTATGAGTACCGAGAAGCTTACGAAGAGGCAGAGCTGCTTGATGATATGTTAGATCAAATCAAGTGGGCAACCGATTATTTTTGAAAGCC   450
            .......310.......320.......330.......340.......350.......360.......370.......380.......390.......400.......410.......420.......430.......440.......450

            ************************************************************************************************.****************.******************************** .**
JN681277    CATACAGGTCCAAATGAATTTTGGGCACAAGTAGGCGATGGGAACGCGGATCATGGCTGGTGGGGTCCAGCAGAAGTGATGCCGATGAACCGGCCGGCATTTAAAATTGATGGACATTGTCCAGGAACAGAAGTAGCCGCCCAAACCACA   600
AF206716.1  CATACAGGTCCAAATGAATTTTGGGCACAAGTAGGCGATGGGAACGCGGATCATGGCTGGTGGGGTCCAGCAGAAGTGATGCCGATGAACCGGCCAGCATTTAAAATTGATGAACATTGTCCAGGAACAGAAGTAGCCGCCCAAACTGCA   600
            .......460.......470.......480.......490.......500.......510.......520.......530.......540.......550.......560.......570.......580.......590.......600

            **************************************:** *****:***************************************************************************************** ************
JN681277    GCCGCTTTAGCAGCAGGTTCAATTATTTTTAAAGAAACTGATGCGCCTTATGCAGCAAAGCTTCTCACCCATGCAAAACAGCTTTATGCATTTGCTGACCAATATCGCGGTGAGTATACAGATTGTGTCACCAATGCGCAGCCATTTTAT   750
AF206716.1  GCCGCTTTAGCAGCAGGTTCAATTATTTTTAAAGAAACAGACGCGCCATATGCAGCAAAGCTTCTCACCCATGCAAAACAGCTTTATGCATTTGCTGACCAATATCGCGGTGAGTATACAGATTGTGTCACCAATGCTCAGCCATTTTAT   750
            .......610.......620.......630.......640.......650.......660.......670.......680.......690.......700.......710.......720.......730.......740.......750

            ** ** *********************************************************************************************************************** ************************
JN681277    AACTCTTGGAGTGGCTATATTGATGAGCTCATTTGGGGTGGAATATGGCTCTACTTGGCGACAAATGACCAGACCTATTTAAACAAAGCACTAAAAGCAGTAGAAGAATGGCCGAAGGATTGGGATTATACGTTTACGATGTCGTGGGAC   900
AF206716.1  AATTCCTGGAGTGGCTATATTGATGAGCTCATTTGGGGTGGAATATGGCTCTACTTGGCGACAAATGACCAGACCTATTTAAACAAAGCACTAAAAGCAGTAGAAGAATGGCCGAAGGATTGGGACTATACGTTTACGATGTCGTGGGAC   900
            .......760.......770.......780.......790.......800.......810.......820.......830.......840.......850.......860.......870.......880.......890.......900

            ***********************************.********************************.******** ************************************************************************
JN681277    AATACCTTTTTTGCTTCGCAAATTTTACTTGCGAGGATCACAAAGGAGAAAAGGTTTATCGAATCGACAGAGCGTAACCTCGATTACTGGTCGACGGGTTTTGTTCAAAATGGAAAAGTAGAAAGAATCACTTATACGCCTGGCGGACTA  1050
AF206716.1  AATACCTTTTTTGCTTCGCAAATTTTACTTGCGAGAATCACAAAGGAGAAAAGGTTTATCGAATCGACGGAGCGTAATCTCGATTACTGGTCGACGGGTTTTGTTCAAAATGGAAAAGTAGAAAGAATCACTTATACGCCTGGCGGACTA  1050
            .......910.......920.......930.......940.......950.......960.......970.......980.......990......1000......1010......1020......1030......1040......1050
```

```
             ******************** *****************************************************************************************************************************
JN681277     GCGTGGTTAGATCAATGGGGGTCACTTCGTTATACAGCAAATGCTGCATTTTTAGCGTTTGTGTACGCAGATTGGGTCTCTGATCAAGAAAAAAAGAATCGATACCAAACGTTTGCGATCAGGCAAACACACTATATGTTAGGGGATAAT  1200
AF206716.1   GCGTGGTTAGATCAATGGGGTTCACTTCGTTATACAGCAAATGCTGCATTTTTAGCGTTTGTGTACGCAGATTGGGTCTCTGATCAAGAAAAAAAGAATCGATACCAAACGTTTGCGATCAGGCAAACACACTATATGTTAGGGGATAAT  1200
             ......1060......1070......1080......1090......1100......1110......1120......1130......1140......1150......1160......1170......1180......1190......1200

             ******************************************************************************************************************************************************
JN681277     CCGCAAAATAGAAGCTATGTCGTTGGGTTTGGCAAAAATCCGCCGATGCATCCACACCATCGAACTGCACATGGCTCATGGTCTAATCAGCTGACAACTCCTTCTTCTCATCGGCACACGCTTTATGGAGCGCTTGTTGGGGGGCCTAAT  1350
AF206716.1   CCGCAAAATAGAAGCTATGTCGTTGGGTTTGGCAAAAATCCGCCGATGCATCCACACCATCGAACTGCACATGGCTCATGGTCTAATCAGCTGACAACTCCTTCTTCTCATCGGCACACGCTTTATGGAGCGCTTGTTGGGGGGCCTAAT  1350
             ......1210......1220......1230......1240......1250......1260......1270......1280......1290......1300......1310......1320......1330......1340......1350

             **.********************************** ************************************************************.*** **********************.************************
JN681277     GCGCAGGATCAGTATACCGATGACATCTCTGACTATGCATCAAACGAGGTAGCAACAGACTATAATGCCGCCTTTACTGGAAATGTAGCCAAAATGGTGCAGTTGTTTGGTCAGGGGCAGTCAAAGCTGCCGAATTTTCCGCCTAAAGAA  1500
AF206716.1   GCACAGGATCAGTATACCGATGACATCTCTGACTATGTATCAAACGAGGTAGCAACAGACTATAATGCCGCCTTTACTGGAAATGTAGCCAAAATGGTACAGCTGTTTGGTCAGGGGCAGTCAAAACTGCCGAATTTTCCGCCTAAAGAA  1500
             ......1360......1370......1380......1390......1400......1410......1420......1430......1440......1450......1460......1470......1480......1490......1500

             ******************************************************************************************************.****************************************.** ***
JN681277     AAAGTGGAAGATGAGTTTTTTGTAGAGGCAGCTGTCATGAGTAACGATACAACATCTACTCAGATCAAAGCCATTCTATACAATCGATCAGGCTGGCCGGCAAGAAGTAGTCAATCACTTTCTTTTAGATATTATGTCAATCTAAGTGAG  1650
AF206716.1   AAAGTGGAAGATGAGTTTTTTGTAGAGGCAGCTGTCATGAGTAACGATACAACATCTACTCAGATCAAAGCCATTCTATACAATCGATCAGGCTGGCCGGCCAGAAGTAGTCAATCACTTTCTTTTAGATATTATGTCAATCTGAGCGAG  1650
             ......1510......1520......1530......1540......1550......1560......1570......1580......1590......1600......1610......1620......1630......1640......1650

             *********************************************** *********************:********************************************************************************
JN681277     ATATTTGCGAAGGGATTCACTGATAAAGATATTCAAGTGACAGCTGTTTACAATGAAGGCGCTTCCTTAACTCCGTTAACGGTGTATGACGCATCATCCCATATCTATTTTACAGAAATCGATTTTACTGGCGTAGCTATTTTTCCAGGA  1800
AF206716.1   ATATTTGCGAAGGGATTCACTGATAAAGATATTCAAGTGACAGCTGTCTACAATGAAGGCGCTTCCTTATCTCCGTTAACGGTGTATGACGCATCATCCCATATCTATTTTACAGAAATCGATTTTACTGGCGTAGCTATTTTTCCAGGA  1800
             ......1660......1670......1680......1690......1700......1710......1720......1730......1740......1750......1760......1770......1780......1790......1800

             ****************************************************************************************************************.**********************************.:*
JN681277     GGCGAATCGCTTCATAAGAAGGAAATACAGTTCCGGTTATCTGCGCCAAATGGTGCGAATATATGGGATGCCTCAAATGATTATTCCTTTCAAGGATTAACATCCAATATGCAGAAAACAGCGAGAATTCCTGTTTTTGATCAAGGTATT  1950
AF206716.1   GGCGAATCGCTTCATAAGAAGGAAATACAGTTCCGGTTATCTGCGCCAAATGGTGCGAATATATGGGATGCCTCAAATGATTATTCCTTTCAAGGATTAACATCCAATATGCAAAAAACAGCGAGAATTCCTGTTTTTGATCAAGGTGAT  1950
             ......1810......1820......1830......1840......1850......1860......1870......1880......1890......1900......1910......1920......1930......1940......1950

             **** *************************
JN681277     TTAGCATTTGGTACGCTTCCGAATAAATAA  1980
AF206716.1   TTAGTATTTGGTACGCTTCCGAATAAATAA  1980
             ......1960......1970......1980
```
